# Editorial: The Dynamic Interface Between Vascular Blood Vessels to Blood Forming Hematopoietic Stem Cells in Health and Disease

**DOI:** 10.3389/fcell.2022.870129

**Published:** 2022-03-04

**Authors:** Tomer Itkin, Delfim Duarte, Diana Passaro

**Affiliations:** ^1^ Division of Regenerative Medicine, Department of Medicine, Weill Cornell Medicine, New York, NY, United States; ^2^ Hematopoeisis and Microenvironments Group, Instituto de Investigação e Inovação Em Saúde (i3S), Universidade Do Porto, Porto, Portugal; ^3^ Department of Onco-Hematology, Instituto Português de Oncologia (IPO)-Porto, Porto, Portugal; ^4^ Department of Biomedicine, Unit of Biochemistry, Faculdade de Medicina da Universidade Do Porto, Porto, Portugal; ^5^ Leukemia and Niche Dynamics Laboratory, Université de Paris, Institut Cochin, Institut National de La Santé et de La Recherche Médicale, Centre National de La Recherche Scientifique, Paris, France

**Keywords:** hematopoeific stem cells, vascular niche, hematopoiesis, endothelial cell (EC), malignant hematopoiesis, developmental hematopoiesis, translational hematopoiesis, bone marrow

## Introduction

**FIGURE 1:**
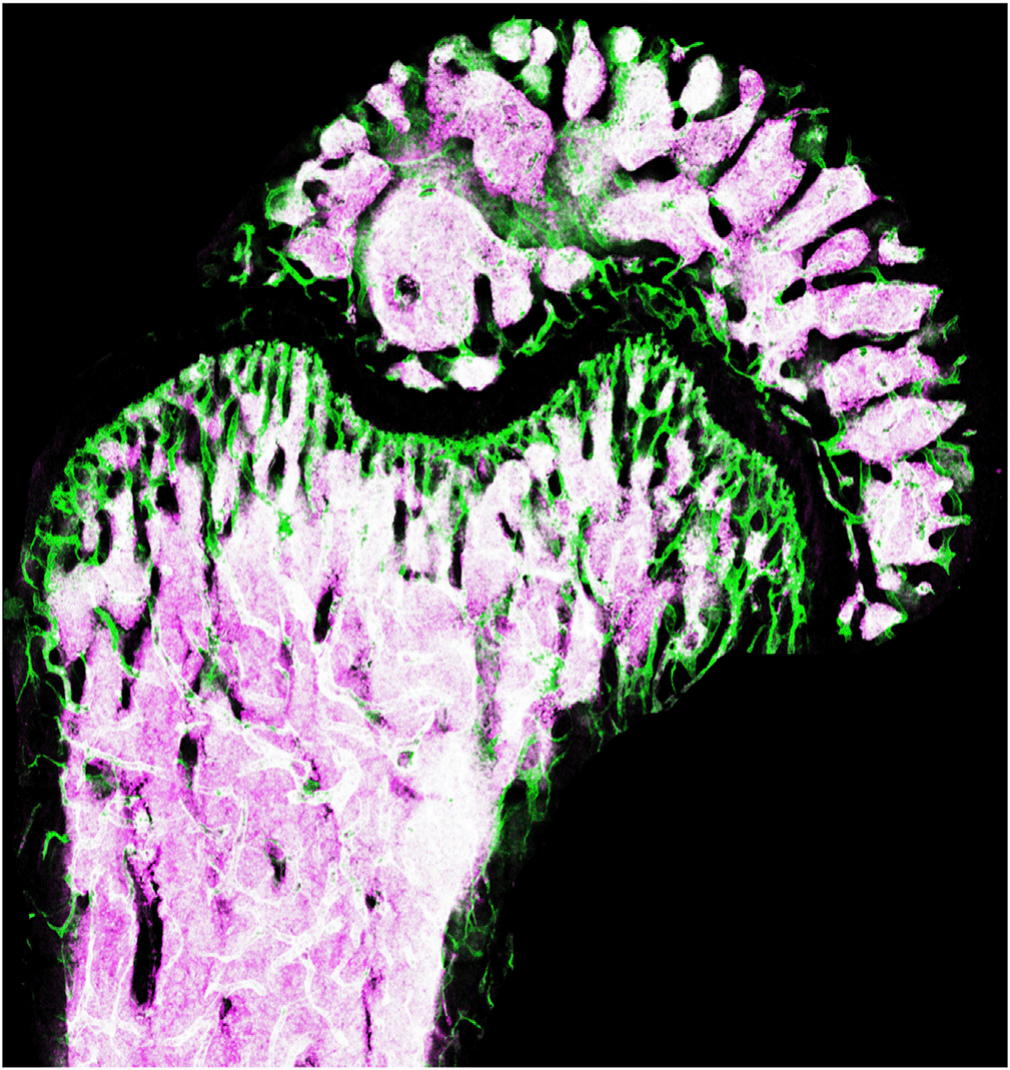
Representative 3D reconstitution image of a femoral bone marrow cross-section from a mouse harboring a leukemic disease of an acute myeloid leukemia (AML) type. Vascular blood vessels are labeled with an antibody for the endothelial marker CD31 (Green), and mouse MLL-AF9 AML cells are labeled by a Tomato reporter (Purple). Image was contributed by the Leukemia and Niche Dynamics Laboratory (D.P.).

In the past 2 decades the concept of stem cell niches, referring to the cell types neighboring stem cells in their microenvironment, have been gaining momentum and biological appreciation (Wu et al., 2021). Vascular blood vessel lining endothelial cells form conduit networks providing tissues with oxygen and nutrients and serve as roads for blood and immune cell trafficking. However, endothelial cells exhibit organotypic specialization and intra-tissual heterogeneity to perform their additional pivotal duty as supporters of organ function during steady state and as facilitators of tissue regeneration and recovery. This support during adulthood is performed by vascular endothelial cells mainly via regulation of their neighboring tissue specific stem cells and thus serve as key components of the stem cell niche (Rafii et al., 2016). In primary hematopoietic organs such as the adult bone marrow, vascular endothelial cells orchestrate numerous functions and developmental fate choice decisions of hematopoietic stem and progenitor cells (Itkin et al., 2016). This calling extends also to malignant conditions under which corrupted vascular niche cells are drafted to augment leukemic cell propagation (Passaro et al., 2017; Duarte et al., 2018) (Figure 1).

Bone marrow endothelial cells associated with malignant leukemic conditions provide hematopoietic tumor cells with sheltering microenvironments accommodating tumor resistance to chemotherapeutic and irradiation therapies and participate in tumor escape from immune surveillance, consenting leukemic relapse. Yet, this tight relationship between blood forming stem cells and vascular blood vessel forming endothelial cells begins at a very early embryonic developmental stage when a special type of hemogenic endothelial cells give rise to primitive and definitive hematopoietic cells responsible for the early waves of hematopoiesis. Moreover, already at this stage neighboring endothelial cells which do not specify to hematopoietic cells provide a supportive niche to this developmental process (Cao et al., 2013). Overall, the gain of insights of this close interaction and crosstalk between endothelial to hematopoietic cells can promote the development and acquisition of therapeutic tools to target leukemic cells, accelerate hematopoietic tissue regeneration and recovery, achieve the generation of blood product from adult endothelial cells, and to *ex vivo* reproduce a vascularized bone marrow on a chip model. This special issue topic, composed of seven review articles, provides an overview of all these themes and aspects ranging from embryonic and adult basic biology studies, to translational studies and recent technological advancements.

## Vascular Developmental Chaperon of Hematopoiesis: From Embryogenesis to Adulthood

The emergence and maintenance of the hematopoietic system relies on the source of a specialized sub-type of hemogenic endothelial cells and at the same time on the incoming guiding signals from neighboring vascular endothelial niche. Hematopoiesis is differentially regulated during development and intimately related to niche specialized endothelial cells in its infancy. Heck et al. provide a comprehensively review of the current evidence on embryonic hematopoiesis and discuss the role of specialized vascular beds in the generation and expansion of hematopoietic stem and progenitor cells (Heck et al., 2020). Studies in different model organisms including the mouse, zebrafish and chicken as well as in human cells are discussed and contribute to a complementary understanding of embryonic hematopoietic development. The authors also highlight areas that will likely contribute to our future understanding of embryonic hematopoiesis, including niche signal integration (to produce a specific hematopoietic output) and transcriptional regulation of heterogenous sub-types of embryonic ECs in their specialization to distinct fates.

During adulthood, hematopoiesis occurs primarily in the bone marrow, where hematopoietic stem and progenitor cells reside in close contact with blood vessels, namely sub-types of arteriolar, sinusoidal, and transitional capillary ECs. In recent years, several studies have better characterized the heterogeneity of the bone vasculature and its functional impact in bone physiology and hematopoiesis. The role of specialized blood vessels in the adult bone is discussed by Hendriks et al., who have previously described the type-H endothelium, an endosteal vascular subtype that is important for bone maintenance and turnover. The authors review the organization of the bone vasculature in detail and discuss the current evidence on how bone marrow vascular niches contribute to bone repair and homeostasis via interaction with hematopoietic and mesenchymal stromal cells (Hendriks and Ramasamy, 2020).

## The Corrupted Bone Marrow Vascular Niche

In addition to the key role of blood vessels in homeostasis, vascular environments are affected and influence disease states. Mosteo et al. focus on the role of the bone marrow vascular niches in myeloid hematologic malignancies, namely acute myeloid leukemia (AML) and myelodysplastic syndromes (MDS) (Mosteo et al., 2021). The authors provide useful information on the vascular niche compartmentalization of the bone marrow and discuss the role of blood vessels in three processes associated with myeloid malignancies: aging, inflammation and clonal hematopoiesis. The authors also provide useful insights on the clinical evidence for and translational potential of analyzing and manipulating the vascular niche in AML and MDS.

Stucker et al. present a larger picture of how the bone marrow vascular niches are remodeled in allostasis (Stucker et al., 2020). The authors start by discussing how environmental signals are integrated in bone endothelial cells and move on to review how bone marrow blood vessels are affected in degenerative diseases such as arthritis, malignancies and by radiotherapy and chemotherapy.

One of the key pathways involved in the interaction between endothelial cells and hematopoiesis is the Notch signaling pathway, which is the subject of analysis by Huang et al. The authors review how Notch is involved in hematopoietic lineage differentiation and in leukemia and attempt to reconcile the contradictory evidence for the importance of Notch signaling in adult HSC maintenance (Huang et al., 2020).

## Recent Technological Advancement in Vascular Niche Dissection and Engineering

Despite being known and studied in depth for decades, only recently the bone marrow microenvironment has been dissected at a single cell level thanks to recent technological advancements, providing the possibilities for dissecting tissues at this resolution. Dolgalev et al., made a concise synopsis of the recent work done in the field, highlighting the molecular features of the bone marrow microenvironment at single cell resolution, emphasizing the most significant differences in tissue dissection for acquisition of cellular material, sequencing and analysis techniques, and computational approaches (Dolgalev and Tikhonova, 2021). The bioinformatic combination of these pulled data reveals interesting features of the vascular endothelial system in the bone marrow, currently classified in three major components which still do not completely recapitulate its full heterogeneity. Further advances combining multi-modal single-cell approaches, such as pairing gene/protein expression data with spatial tissue context and *in vivo* imaging will deepen our understanding of the vascular niche and resolve standing questions.

As recent technological advances have allowed a thorough characterization of the cellular and matrix components of the vascular bone marrow niche, modelling capabilities have followed up with the aim of reproducing 3D bone marrow-like structures, which can support normal and malignant hematopoiesis either *in vitro* or *in vivo*. Several experimental systems have been implemented and have displayed the feasibility of bioengineering bone marrow-like tissues, supported by cells of mesenchymal origin. Despite being largely disregarded in the first models, vascular endothelial cells have gradually been included in these constructs. Bessy and colleagues have reviewed the field of bone-tissue bioengineering with a particular focus on vascular implementation and integration (Bessy et al., 2021). The field still faces the big challenge of transitioning from experimental models to translational research. Nevertheless, some studies have recently reached the clinical stage and pave the way for the use of vascularized BM bioengineered devices for regenerative medicine.

## Conclusion

In summary, our research topic brings forward the most recent advancements in the research area of the hematopoietic stem cell niche, highlighting the fact that endothelial and blood cells are bound together for life from the early time points of embryogenesis, throughout adulthood and aging, during homeostasis and disease, tightly regulating and affecting each other’s fate choice decisions. This multidisciplinary field of studies combining hematopoiesis and vascular biology, is rapidly moving forward, thanks to recent technological advances, gaining insights and major relevance to hematological diseases. As it gains momentum, more and more basic scientists as well as clinicians begin to appreciate the translational potential and the novel therapeutic approaches that can be developed based on the basic biological findings presented in this review collection. We predict that in the upcoming years this research topic will continue to rapidly evolve and attract scientific and clinical interest leading to major discoveries further contributing to better biological understanding of intra-tissual cellular interactions and advancing clinical approaches to treat disease.
